# Болезнь Иценко-Кушинга у мальчика 15 лет, манифестировавшая в препубертатном возрасте

**DOI:** 10.14341/probl13547

**Published:** 2025-09-14

**Authors:** Ю. Л. Скородок, И. Ю. Иоффе, Е. В. Плотникова, Н. Ю. Калинченко, И. И. Нагорная, А. В. Кожевникова, Н. В. Казаченко, О. В. Филиппова

**Affiliations:** Санкт-Петербургский государственный педиатрический медицинский университет; Saint-Petersburg state pediatric medical university; Национальный медицинский исследовательский центр эндокринологии им. академика И.И. Дедова; I.I. Dedov Endocrinology Research Centre; Детский городской многопрофильный клинический центр высоких медицинских технологий им. К.А. Раухфуса; Children’s city multidisciplinary clinical center of high medical technologies named after K. A. Rauhfus

**Keywords:** гиперкортицизм, болезнь Иценко-Кушинга, малый дексаметазоновый тест, селективный забор крови из нижних каменистых синусов, трансфеноидальная аденомэктомия, дети, hypercortisolism, Cushing’s disease, overnight dexamethasone test, selective blood sampling from the inferior petrosal sinus, transsphenoidal adenomectomy, children

## Abstract

Cushing’s disease (CD) is the leading form (75–80%) of the endogenous hypercortisolism (EH) in adolescents. Despite the obvious clinical manifestations, the diagnosis of CD is complicated because of the need of several research methods, the risk of false-positive and false-negative results, difficulties in interpretation and the low percentage of MR-imaging in children. The present patient had noted excessive weight gain since the age of 10 years, a decrease in growth rate was detected retrospectively, absolute short stature developed by the age of 14.5 years. An examination at the age of 15 revealed an inversion of the circadian cortisol rhythm (9:00 378.4 nmol/l, 23:00 598.9 nmol/l), an increase in the cortisol level in saliva at 23:00 (20.32 nmol/l) and excretion in urine (981.5 mcg/day). The overnight dexamethasone test (ODT) was positive (cortisol 44.26 nmol/l), ACTH was in the “gray zone” (22.19 pg/ml). MRI with contrast showed signs of a heterogeneous pituitary gland structure. When patient was re-examined at 15.5 years the inversion of the circadian rhythm of cortisol was accompanied by absolute hypercortisolemia (9:00 843.4 nmol/l, 23:00 929.4 nmol/l), ODT became negative (cortisol 235 nmol/l), ACTH level remained in the “gray zone” (25.1 pg/ml). MRI with contrast showed pituitary adenoma 6×4 mm. An ACTH level gradient between the left inferior petrosal sinus and peripheral blood of 13.3 confirmed CD. After transsphenoidal adenomectomy, a cortisol level of 39.4 nmol/l indicated remission with a low risk of relapse; subsequently a reverse development of EH symptoms were noted. Postoperative diabetes insipidus and primary hypothyroidism required replacement therapy.

## АКТУАЛЬНОСТЬ

Болезнь Иценко-Кушинга (БИК) — редкое нейроэндокринное заболевание, возникающее вследствие гиперсекреции адренокортикотропного гормона (АКТГ) аденомой гипофиза и являющееся ведущей формой (75–80%) эндогенного гиперкортицизма (ЭГ) у подростков и взрослых [1][2] Распространенность БИК составляет 39,1:1000000 человек, у детей — 0,12–0,24:1000000 [3][4].

Несмотря на характерные клинические проявления, диагностика БИК в детском возрасте затруднительна в связи с редкостью встречаемости данного состояния, медленным нарастанием клинической симптоматики. Отличительной клинической особенностью БИК в детском возрасте является снижение скорости роста с развитием низкорослости при поздней постановке диагноза [5]. По данным литературы, от появления первых признаков заболевания до постановки диагноза проходит от 6 месяцев до 6,5 года [1][6][7]. Для корректной диагностики требуется целый ряд исследований [8], при проведении которых имеется риск ложноположительных и ложноотрицательных результатов, а также существуют затруднения в интерпретации тестов. Кроме того, эффективность визуализации кортикотропином у детей при МРТ с контрастированием составляет около 50% [4]. В случае, если аденома не выявляется или ее размеры менее 6 мм, рекомендуется проведение селективного забора крови из нижних каменистых синусов (НКС), возможного только в специализированном учреждении [3].

## ОПИСАНИЕ СЛУЧАЯ

Пациент Д., 2008 г. р. обратился в городской детский эндокринологический центр в возрасте 14 лет 4 месяцев с жалобами на прогрессирующую прибавку массы тела на фоне соблюдения диеты и активных занятий спортом.

Масса тела при рождении — 3900 г, рост — 53 см, оценка по шкале Апгар 8/9 баллов. Ранний анамнез без особенностей. В возрасте 7 лет перенес серозный менингит. Семейный анамнез по ожирению, сахарному диабету не отягощен. Рост матери — 164 см, отца — 184 см, старшего брата — 187 см.

С 10 лет прогрессирующий набор массы тела несмотря на занятия спортом (тренажерный зал, лыжи). Впервые обследован в 10 лет 7 месяцев: рост — 147 см (SDS +0,98), масса тела — 49 кг, индекс массы тела (ИМТ) — 22,7 кг/м² (SDS +2,33). Глюкоза плазмы (ГП) натощак 4,7 ммоль/л (4,4–6,1), ТТГ — 0,65 мМЕ/л (0,51–4,3), свободный Т4 (свТ4) — 13,3 пмоль/л (12,6–21,0), антитела к ТПО и ТГ отрицательные, пролактин — 193,1 мМЕ/л (86–324). Рекомендованы диета и физические нагрузки, на фоне которых отмечалось снижение массы тела.

В возрасте 12 лет 3 месяцев появились жалобы на головные боли, периодическую утомляемость, эпизодическое повышение артериального давления (АД) до 122/78–126/83 мм рт.ст., обморочные состояния на фоне голодания, психоэмоционального напряжения. При обследовании у кардиолога патология сердечно-сосудистой системы исключена. В 12 лет 6 месяцев рост — 148,5 см (SDS -0,56), масса тела — 59,6 кг, ИМТ — 27,0 кг/м² (SDS +2,5). При ретроспективном анализе обратило на себя внимание выраженное снижение скорости роста до 0,7 см/год (SDS -4,03). АД — 110/70 мм рт.ст. Половое развитие по Tanner PII, объем тестикул по Prader d=2 ml, s=3 ml. Выявлена нарушенная толерантность к глюкозе (НТГ): ГП натощак 5,3 ммоль/л (<6,1), через 2 часа 9,7 ммоль/л (<7,8) при нормальном содержании инсулина в крови 15,6 мкЕд/мл (2,0–25,0). Первичный гипотиреоз исключен (ТТГ — 1,33 мМЕ/л; норма 0,51–4,3). Уровни ЛГ<0,1 МЕ/л (0,02–4,9), ФСГ — 0,59 МЕ/л (0,26–3,2) и тестостерона (Т) 1,4 нмоль/л (1,03–5,19 нмоль/л) соответствовали возрастной норме. Костный возраст 10,5–11 лет по Greulich. При УЗИ органов брюшной полости выявлены диффузные изменения поджелудочной железы. Диагностированы ожирение 2 степени, НТГ, синдром неправильного пубертата (СНП). Рекомендованы диета, физические нагрузки, метформин по 500 мг 2 раза в день, повторное обследование через 6–12 месяцев. Пациент получал лечение в течение года, затем самостоятельно его отменил.

В 14 лет 4 месяца рост — 151 см (SDS -1,8), скорость роста низкая (1,5 см/год; SDS -2,8), масса тела — 59,9 кг, ИМТ — 26,27 кг/м² (SDS +2,01). Половое развитие по Tanner PIV, объем тестикул по Prader d=s=8 ml. Костный возраст 14,5–15 лет по Greulich. Данных за нарушение углеводного обмена не получено (ГП натощак — 4,9 ммоль/л (<6,1), через 2 часа после нагрузки — 5,4 ммоль/л (<7,8)), исключены заболевания щитовидной железы (ТТГ — 0,97 мМЕ/л (0,51–4,3), свТ4 — 16,02 пмоль/л (12,6–21,0), антитела к ТПО и ТГ отрицательные, УЗИ в норме). Концентрация ИФР-1 резко снижена (109 нг/мл; SDS <-2). Выявлено умеренное повышение утреннего уровня кортизола (626,7 нмоль/л; норма 133–537) в сочетании с высоким содержанием дегидроэпиандростерона сульфата (866,2 мкг/дл, норма 70,2—492), прегненолона (13 нг/мл, норма 0,1–3,4) и нормальными концентрациями андростендиона (2,194 нг/мл, норма 0,33–3,3) и 17-гидроксипрогестерона (1,0 нг/мл, норма 0,2–3,1). Несмотря на прогрессирование полового оволосения базальные уровни ЛГ<0,1 МЕ/л (0,2–7,0), ФСГ — 0,47 МЕ/л (1,8–9,2), Т — 0,537 нг/мл (1,8–7,63) соответствовали допубертатным значениям, отсутствовало повышение уровня ЛГ в ходе теста с трипторелином (0,931 МЕ/л (>8)). Трехдневный тест с ХГ положительный (9,515 нмоль/л, норма >3,5). Концентрации АМГ 12,5 нг/мл (4,6–22,8) и ингибина В 114,4 пг/мл (62–338) в пределах возрастной нормы, отношение ингибин В/АМГ 9,152 (нет данных о референтном интервале). При МРТ головного мозга и хиазмально-селлярной области с контрастированием выявлены признаки неоднородной структуры гипофиза. Диагностированы ожирение 1 степени, НТГ (от 2021 г.), низкорослость умеренной степени, гипогонадотропный гипогонадизм (ГГ). Рекомендован курс смеси эфиров тестостерона 100 мг 1 раз в 28 дней №3.

В 15 лет 3 месяца (рис. 1), через 3 месяца после окончания курса терапии смесью эфиров тестостерона рост — 151 см (SDS -2,41), скорость роста 0 см/год, масса тела 64 кг; +4 кг/год; ИМТ 28,1 кг/м² (SDS +2,17), подкожно-жировая клетчатка распределена по центрипетальному типу. Кожные покровы истончены, с мраморностью, багровый румянец, гипертрихоз, в подмышечных областях acantosis nigricans; стрии отсутствуют. АД — 120/75 мм рт.ст. Половое развитие по Tanner PIV, объем тестикул по Prader d=s=8 ml. Данных за нарушение углеводного обмена нет (ГП натощак — 5,2 ммоль/л (<6,1), через 120 минут — 4,99 ммоль/л (<7,8), уровень HbA1c — 5,23% (<5,5)). Выявлены инверсия суточного ритма кортизола (в 09:00 — 378,4 нмоль/л, норма 133–537, в 23:00 — 598,9 нмоль/л, норма ≤50% от утреннего), повышение уровня кортизола в слюне в 23:00 (20,32 нмоль/л; точка разделения 9,4) и экскреции с мочой (981,5 мкг/сут; норма <403). Кортизол в ходе ночного дексаметазонового теста (МДТ) 44,26 нмоль/л (<50). АКТГ — 22,19 пг/мл (7,2–63,3). По результатам суточного мониторирования АД выявлена дневная систоло-диастолическая артериальная гипертензия, стабильная систолическая, лабильная диастолическая. При ФГДС отмечался неэрозивный рефлюкс-эзофагит, очаговый гастрит, дуодено-гастральный рефлюкс. В ходе КТ исключены объемные образования органов брюшной полости и забрюшинного пространства. Рентгеновская денситометрия позвоночника (L2-L4) выявила снижение минеральной плотности костной ткани (Z-критерий -2,4 SD). Диагностирован АКТГ-зависимый эндогенный гиперкортицизм — болезнь Иценко-Кушинга без визуализации аденомы. Осложнения: вторичный дефицит гормона роста. Симптоматический гипогонадизм. Артериальная гипертензия. Остеопения. Сопутствующие: НТГ (от 2021 г). Неэрозивный рефлюкс-эзофагит, очаговый гастрит, дуодено-гастральный рефлюкс. Для дальнейшего обследования и лечения направлен в ФГБУ «НМИЦ эндокринологии» Минздрава России.

**Figure:**
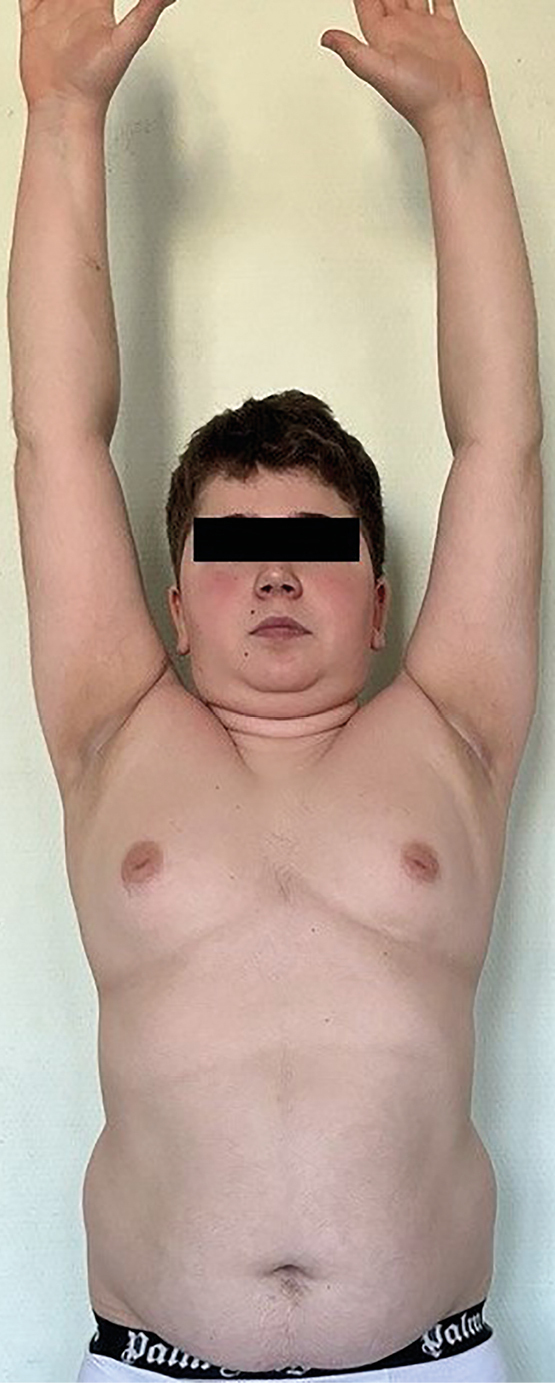
Рисунок 1. Пациент, 15 лет 3 мес.

В ФГБУ «НМИЦ эндокринологии» в возрасте 15 лет 5 месяцев рост — 151 см, масса тела — 67,5 кг (+3,5 кг за 2 месяца) выявлены гиперкортизолемия и инверсия суточного ритма кортизола и АКТГ: кортизол в 09:00 — 843,4 нмоль/л (77,0–630,0), в 23:00 — 929,4 нмоль/л (64,0–327,0), АКТГ в 09:00 — 25,1 пг/мл (7,2–63,3), в 23:00 — 53,4 пг/мл (2,0–25,5). Отмечались дальнейшее повышение экскреции кортизола (2749,5 нмоль/л; норма 100,0–329,0) и уровня кортизола слюны в 23:00 (72,0 нмоль/л; точка разделения 9,4), отрицательный МДТ (кортизол 235 нмоль/л; норма <50). При МРТ с контрастированием выявлена аденома гипофиза 6×4 мм. Для подтверждения центрального генеза гиперкортицизма проведен селективный забор крови из каменистых синусов: градиент уровня АКТГ между левым нижним каменистым синусом (800 пг/мл) и периферической кровью (60 пг/мл) составил 13,3 (>2 для БИК). Подтвержден диагноз БИК вследствие аденомы гипофиза, выполнена транссфеноидальная аденомэктомия. Послеоперационный уровень кортизола в 09:00 — 39,4 нмоль/л, АКТГ — 6,4 пг/мл. На вторые сутки начата заместительная терапия гидрокортизоном 25 мг/сут (15 мг/м²; с постепенным снижением), в связи с полиурией и тенденцией к гипернатриемии (натрий до 147,8 ммоль/л) — десмопрессином 0,025–0,05 мг/сутки.

Через 2 недели после хирургического лечения в связи с жалобами на выраженную слабость, сонливость, тошноту обследован, выявлены гиперферментемия (АЛТ — 150,4 Ед/л, АСТ — 63,4 Ед/л) и синдром первичного гипотиреоза (ТТГ — 73,97 мМЕ/л, свТ4 — 11,36 пмоль/л (12,6–21,0)) без признаков АИТ (антитела к ТПО не обнаружены, УЗИ щитовидной железы в норме). Начато лечение левотироксином натрия в стартовой дозе 25 мкг/сутки, урсодезоксихолевой кислотой, на фоне которого самочувствие улучшилось. Через 6 месяцев после операции (рис. 2) рост — 156 см (SDS -2,16), скорость роста — 10 см/год (SDS +2,25), масса тела — 55 кг, ИМТ — 22,6 кг/м² (SDS +0,74). АД — 107/73 мм рт.ст., ЧСС — 80 уд/мин. Половое развитие по Tanner PIV, объем тестикул по Prader d=s=20 мл. АЛТ — 26,9 Ед/л, АСТ — 24,8 Ед/л, ТТГ — 4,8 мМЕ/л (левотироксин натрия 75 мкг/сутки), ЛГ — 5,5 МЕ/л, ФСГ — 4,02 МЕ/л, Т — 6,14 нг/мл. Диурез — 3150 мл, относительная плотность мочи на фоне терапии 1007–1018 (десмопрессин — 0,075 мг/сутки).

**Figure:**
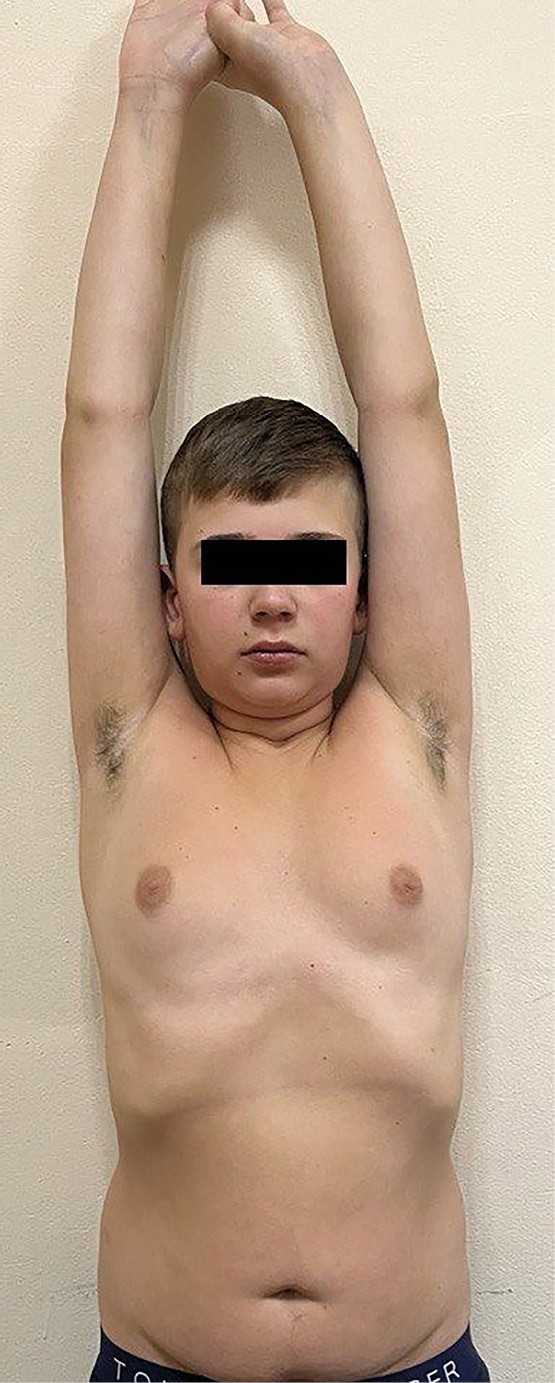
Рисунок 2. Пациент 15 лет 11 мес (6 месяцев после аденомэктомии, 6 дней после возвращения с курорта).

## ОБСУЖДЕНИЕ

Первой жалобой у описываемого пациента была избыточная прибавка массы тела, появившаяся за 5 лет до установления диагноза. Это особенно беспокоило пациента и его семью в связи с отсутствием ожирения в семейном анамнезе и активным образом жизни. Ретроспективная оценка позволила установить, что снижение скорости роста отмечалось параллельно с избыточными прибавками массы тела. Абсолютный рост в течение 2 лет оставался в пределах средних значений, хотя ∆SDS роста составила -0,77/год. Лишь спустя 4 года от начала заболевания задокументирована низкорослость легкой степени, 5 лет — умеренной. По данным литературы, снижение скорости роста — один из наиболее значимых и ранних проявлений ЭГ у детей, как правило предшествующий появлению других симптомов заболевания. Чем младше ребенок и больше длительность заболевания, тем значительнее задержка роста [2][9]. По данным Н.А. Стребковой и соавт. (2009 г.), у детей с БИК на момент диагностики SDS роста составлял от -0,7 до -4,2 [10].

Такие симптомы ЭГ, как характерное распределение подкожного жирового слоя, матронизм, гипертрихоз, были выявлены у описываемого пациента только спустя 5 лет от начала заболевания, а атрофические стрии (и любые виды стрий), один из типичных симптомов ЭГ у взрослых, отсутствовали. Аналогичные наблюдения представлены М.А. Тюльпаковым и соавт. (2022 г.) [4]. Обращало на себя внимание, что степень выраженности лобкового оволосения по Tanner (II) не соответствовала объему тестикул (d=s=2 ml), что связано со стимуляцией АКТГ-адреналового андрогенеза. Артериальная гипертензия, характерная более чем для 70% детей с БИК [11], у нашего пациента была выявлена только по результатам инструментального исследования (суточное мониторирование артериального давления).

Умеренное повышение утреннего уровня кортизола было впервые зафиксировано в 14 лет 4 месяца, то есть через 4 года после появления первых симптомов, и стало поводом для инициации полноценного лабораторного обследования. Диагноз «ЭГ» подтвержден нарушением суточного ритма кортизола крови, повышением его уровня в слюне в 23:00 и экскреции кортизола с мочой. Обращает на себя внимание, что при первичном обследовании результат МДТ был положительным (несмотря на его высокую чувствительность по данным литературы) [3], а уровень АКТГ — не диагностическим [8]. Параллельно с прогрессированием заболевания и утяжелением клинической картины отмечалось соответствующее ухудшение лабораторных показателей (МДТ стал отрицательным, а уровень АКТГ — диагностическим).

В послеоперационном периоде у пациента развился несахарный диабет. По данным литературы, это осложнение (чаще транзиторного характера) встречается в 13–30% случаев лечения селлярных и супраселлярных опухолей, при этом самый высокий риск отмечается после резекции краниофарингиомы, кисты кармана Ратке и микроаденом [12][13].

Центральный гипокортицизм, развившийся после операции, требует лечения, но может носить транзиторный характер. По данным Q. Cui и соавт. (2023 г.), ось «гипоталамус-гипофиз-надпочечник» восстановилась у 73,6% пациентов с БИК в течение 2 лет после хирургического лечения, а среднее время восстановления составило 12 месяцев. Более высокую вероятность перманентного гипокортицизма имели пациенты с недостаточностью других гормонов аденогипофиза [14].

Через 2 недели после операции у пациента был диагностирован первичный манифестный гипотиреоз. В исследовании B. Xiang и соавт. (2019 г.) концентрации ТТГ выше референтного диапазона отмечались лишь у двух пациентов из 102 и нормализовались через 1 год. Авторы связывают выявленные изменения со снижением уровня кортизола в крови после операции и прекращением его ингибирующего действия на секрецию ТТГ [15]. Имеются сведения об ассоциации гиперкортицизма со снижением концентрации тироксин-связывающего глобулина [16]. Нормализация уровня этого белка после проведенного лечения может объяснять временное снижение уровней свТ3 и свТ4. Кроме того, по данным H. Niepomniszcze и соавт. (2002 г.), пациенты с гиперкортицизмом демонстрируют высокую распространенность заболеваний щитовидной железы после хирургического лечения в связи со снижением сдерживающего влияния избытка глюкокортикоидов на течение аутоиммунных процессов [17]. Отсутствие антител и невысокая потребность в левотироксине в нашем случае косвенно указывает на связь выявленного гипотиреоза с операцией и, возможно, транзиторный его характер, что можно будет установить только при последующем мониторинге.

В послеоперационном периоде отмечалось прогрессирование полового развития: увеличение объема тестикул по Prader, повышение уровней гонадотропных гормонов и Т. По данным H. Zheng и соавт. (2022 г.), гипогонадизм (ГГ) наблюдался у 75% пациентов с БИК на момент постановки диагноза. При этом 80% пациентов достигали эугонадизма в течение 12 месяцев после операции, из них большая часть — в течение 3 месяцев [18].

## ЗАКЛЮЧЕНИЕ

Поскольку снижение скорости роста предшествовало развитию абсолютной низкорослости как в описанном случае, так и по литературным данным, оценка этого показателя показана всем пациентам с избыточным весом.

Нормальная супрессия кортизола в ходе малого дексаметазонового теста не исключает диагноз эндогенного гиперкортицизма при наличии двух других лабораторных маркеров заболевания.

Сложностью диагностики болезни Иценко-Кушинга в данном случае явилось отсутствие визуализации аденомы (МРТ) при первичном обследовании, что потребовало селективного забора крови из нижних каменистых синусов.

После транссфеноидальной аденомэктомии, несмотря на малые размеры образования, развился перманентный несахарный диабет. В послеоперационном периоде также манифестировал первичный гипотиреоз неиммунного генеза, характер и причина которого остаются неясными.

## ДОПОЛНИТЕЛЬНАЯ ИНФОРМАЦИЯ

Источники финансирования. Работа выполнена по инициативе авторов без привлечения финансирования

Конфликт интересов. Авторы декларируют отсутствие явных и потенциальных конфликтов интересов, связанных с содержанием настоящей статьи.

Участие авторов. Все авторы одобрили финальную версию статьи перед публикацией, выразили согласие нести ответственность за все аспекты работы, подразумевающую надлежащее изучение и решение вопросов, связанных с точностью или добросовестностью любой части работы.

Согласие пациента. Пациент добровольно подписал информированное согласие на публикацию персональной медицинской информации в обезличенной форме, фотографий в журнале «Проблемы эндокринологии»

Благодарности: выражаем благодарность врачу-нейрохирургу ГНЦ РФ ФГБУ «НМИЦ эндокринологии» Минздрава России, кандидату медицинских наук Азизяну Вилену Мироновичу за высокоэффективную хирургическую помощь пациенту.
